# Vertiginous epilepsy in the pediatric population

**DOI:** 10.3389/fneur.2024.1403536

**Published:** 2024-07-05

**Authors:** Alexandra M. Wood, Adam Thompson-Harvey, Bradley W. Kesser

**Affiliations:** ^1^Department of Neurology, Division of Pediatric Neurology, University of Virginia, Charlottesville, VA, United States; ^2^Department of Otolaryngology and Head and Neck Surgery, Division of Otology and Neurotology, University of Virginia, Charlottesville, VA, United States

**Keywords:** vertigo, dizziness, epilepsy, vestibular system, pediatrics, vestibular migraine, benign paroxysmal vertigo of childhood (BPVC)

## Abstract

Vertiginous epilepsy (VE) is a rare and underrecognized epilepsy subtype in the pediatric population. Vertiginous symptoms are the sole or predominant feature, arise from the vestibular cortex, and seizures are usually brief. The incidence is estimated to be between six and 15 percent of pediatric patients presenting with dizziness. Diagnosis is often delayed for many years following the onset of symptoms, as there are no widely accepted diagnostic criteria. Diagnostic work-up should include a detailed history, physical exam, EEG, and brain imaging with MRI. Vestibular testing is helpful if peripheral vestibulopathy is suspected. Vertiginous epilepsy can have many possible causes, but a large majority are idiopathic or suspected to be genetic. Most patients with vertiginous epilepsy achieve seizure freedom with anti-seizure medications.

## Highlights

- VE originates from the known vestibular cortex, specifically parts of the parietal operculum and the posterior insular; symptoms arise from involvement of the multi-sensory vestibular cortical network.
- While vertigo is a hallmark symptom, VE can be differentiated from non-epileptic vestibular disorders (RVC, VMC, and vestibular paroxysmia) based on lack of migraine history/features, brief vestibular symptom duration, and alterations in consciousness.
- Work-up should include a detailed history, EEG, and imaging, as ruling out other causes is crucial for diagnosis.
- VE typically responds well to anti-epileptic medication, with the choice depending on seizure type and individual factors.

## Introduction

In 1977, pediatric neurologists Eviatar and Eviatar reported epilepsy as the most common cause of vertigo in children referred to their clinic (1). Benign Paroxysmal Vertigo of Childhood (BPVC) and Vestibular Migraine of Childhood (VMC) are now widely recognized as the most common causes of pediatric vertigo/dizziness (2–5). On the other hand, vertiginous epilepsy (VE) is an under-recognized cause of dizziness in children. Vertiginous epilepsy (VE) is a specific epilepsy characterized by focal seizures with vestibular symptoms as either the sole or predominant feature. A systematic review by Tarnutzer and colleagues reported children being diagnosed with vertiginous epilepsy 8.7 times more than adults (6). Although isolated vestibular symptoms are rare in those with epilepsy (<0.5%), the current estimates of the incidence of VE among those presenting with dizziness in the pediatric population range between 6.6 to 15% (7–9), likely secondary to the high incidence of epilepsy in pediatric patients (10–12).

Vertiginous epilepsy (VE), also referred to as epileptic vertigo or dizziness (EVD), vestibular epilepsy, or vestibular seizures can be challenging to diagnose, as symptoms of the seizure can manifest as an initial aura of focal or generalized seizures or as an isolated symptom. Hewett and colleagues reported an average of a four-year delay in VE diagnosis following the onset of symptoms (13). Diagnosing VE in the pediatric population is challenging owing to a lack of clear communication skills among children (and their parents) to describe symptoms and difficulty performing vestibular function tests. Such challenges require a unique diagnostic approach. This mini-review provides an up-to-date overview for understanding, diagnosing, and managing VE in the pediatric population.

## Pathophysiology

**Figure 1:**
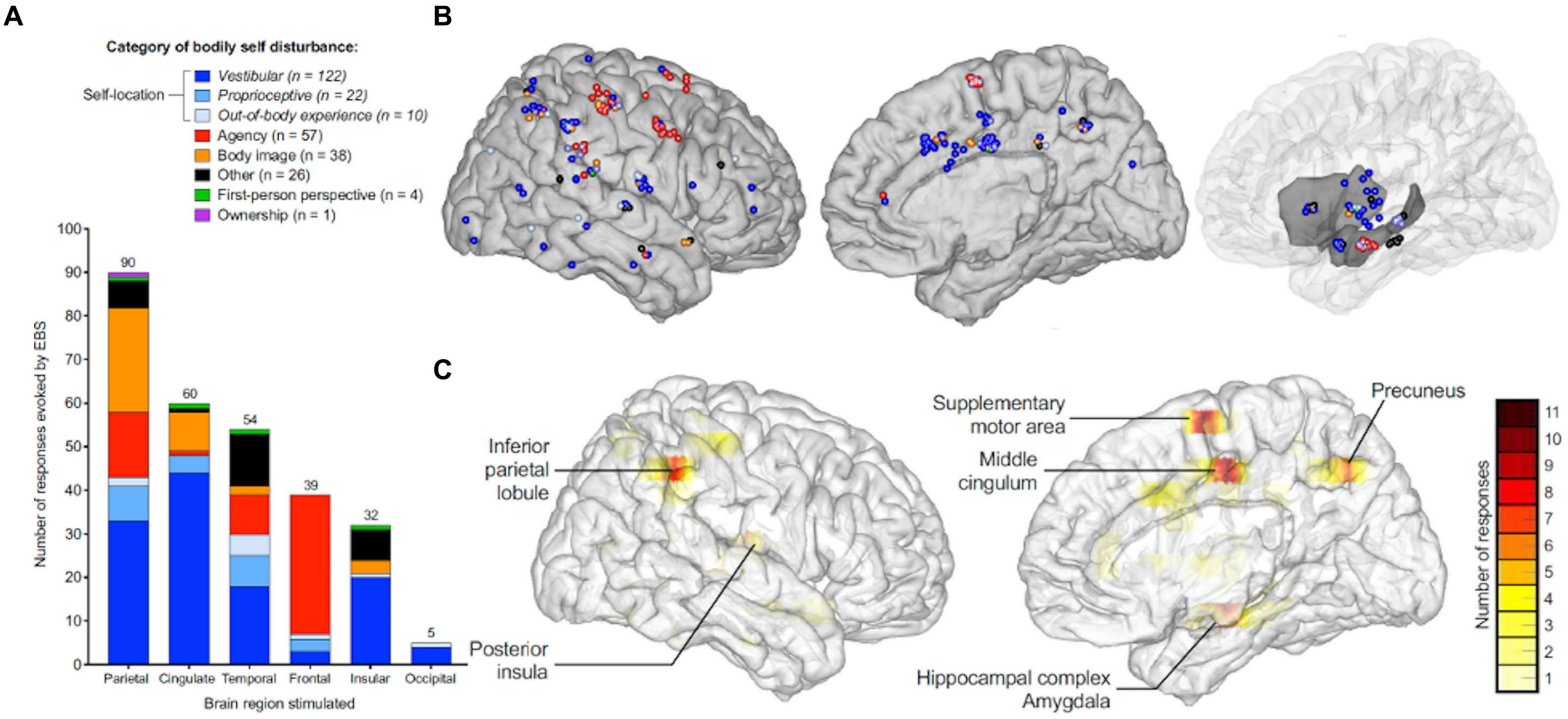
Anatomic and symptom distribution and representation of the areas associated with the multi-sensory vestibular cortical network based on a systematic review of localization studies using intracranial electrical brain stimulation evoking vestibular symptoms. **(A)** Distribution of reported frequencies in various bodily self disturbances based on stimulated brain region. **(B)** Color-coded sites within the right cerebral hemisphere indicating the category of bodily self disturbance upon iEBS. **(C)** Color-coded density maps representing the six main cortical areas eliciting bodily self disturbances on iEBS, based on frequency of response. iEBS= Intra-cranial electrical brain stimulation. Adapted with permission from “Neural bases of the bodily self as revealed by electrical brain stimulation: A systematic review” by Dary et al. (29) in Human Brain Mapping, licensed under CC-BY-NC-ND 4.0 license, published by Wiley Periodicals, LLC.

While focal seizures arising from any brain region may be accompanied by vestibular symptoms if the epileptogenic or symptomatic zone involves the vestibular cortex or its pathways, in VE, the origin of the seizure is presume to be located in the cortex responsible for vestibular semiology (14, 15). Work by Penfield and Jasper (16) as well as Kahane et al. (17) established that vestibular sensations could arise from electrical stimulation of the lateral temporo-parietal cortex (i.e., temporo-peri-Sylvian vestibular cortex). More recently, the vestibular cortex has been described as a distinct network involved in processing multi-sensory vestibular information (15), whereby symptoms may arise from a single region within the vestibular cortex or from the spread of electrical activity to or from nearby regions. While the vestibular cortex has been investigated using primate models (18–24), studies using electrical cortical stimulation and neuroimaging have mapped the human vestibular cortical network to include the parietal operculum and posterior insula primarily (25–28). Additionally, a wide array of vestibular symptoms have been elicited by electrical stimulation of subsites within the temporal and parietal areas, including the superior temporal gyrus, the angular gyrus, the supramarginal gyrus, the hippocampus, the cingulate gyrus, and the precuneus (Figure 1).

## Diagnostic approach

### Presentation

**Figure 2:**
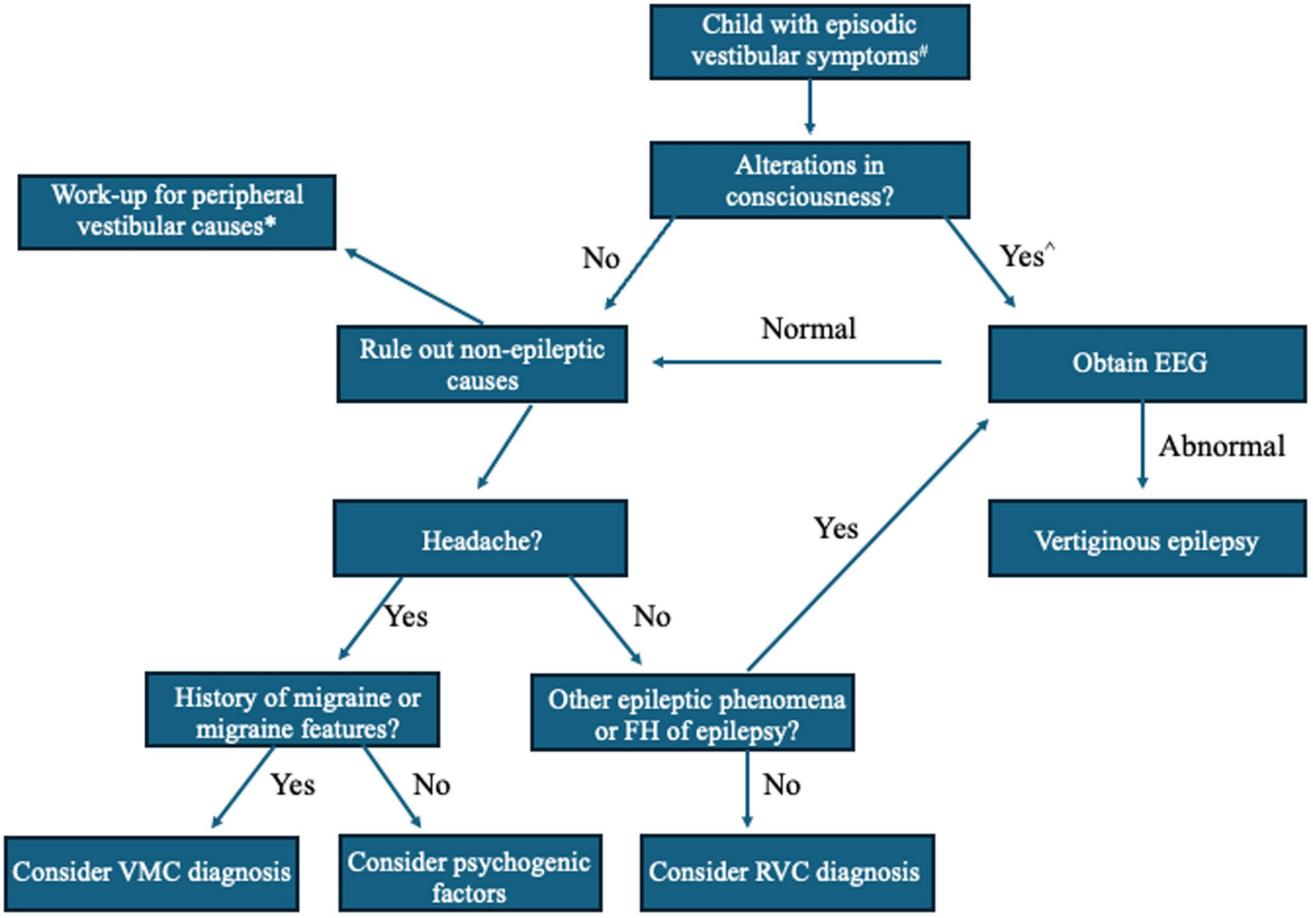
A simplified algorithm for evaluation of vertigo in children suspected of having vertiginous epilepsy (VE). Adapted from Batu et al. *European Journal of Pediatric Neurology*. 2015 (8), Dasgupta et al. *Current Treatment Options in Neurology.* 2020 (33), and Peterson and Brodsky, *Curr Opin Otolaryngol Head Neck Surg.* 2022 (34). # = If focal neurological findings or history of head trauma, obtain brain MRI or head CT. ^ = Perform orthostatic blood pressure measurements, consider ophthalmological exam and electrocardiogram (ECG) or bloodwork (e.g., thyroid function, complete blood cell count, electrolytes). * = Peripheral vestibular disorder work-up should focus on duration and triggers of vestibular episodes, otologic, neurologic, and oculomotor examinations (i.e., rule out middle ear disease, third window phenomena, or nystagmus), and consider vestibular function tests based on age and test tolerance, such as video head-impulse (vHIT), rotatory chair (ROT), and vestibular-evoked myogenic potential (VEMP) testing. EEG, electroencephalography; FH, family history; VMC, vestibular migraine of childhood; RVC, recurrent vertigo of childhood.

Before referral to Neurology, children and adolescents with dizziness are often seen in the primary care setting or evaluated by a subspecialist, such as a Pediatric Otolaryngologist or Neurotologist (30–32). As a common chief complaint, “dizziness” is a blanket term for altered spatial orientation that can include vertigo, disequilibrium, imbalance confusion, lightheadedness, or spatial discomfort and should be clarified by the clinician. “Vertigo,” a hallmark symptom of vestibular dysfunction, is defined as experiencing rotatory sensations (self-motion or visual) or other illusions of motion, such as tilting, floating, rocking, rolling, or a sense of falling forward or backward. Vertigo is the sole or primary symptom in VE, lasting seconds to minutes, though the pediatric patient may present with other symptoms (6). Batu et al. (8) reported the following associated symptoms in those diagnosed with VE (15%) among their retrospective analysis of 100 children presenting to their Pediatric Neurology clinic: headache (20%), nausea (26.6%), vomiting (20%), pallor (13.3%), staring (13.3%), and blackout (6.6%). Among these symptoms, staring was the only significant differentiating symptom between VE and other common causes of vertigo in children, such as BPVC, VM, and psychogenic vertigo (8). Due to these findings, it has been suggested that any changes in cognitive function or alterations in consciousness that accompany vertigo in the pediatric population should prompt the clinician to investigate for possible VE (Figure 2) (8, 35). Positional vertigo or hearing loss do not support the diagnosis of VE though auditory hallucinations (e.g., pure tone tinnitus, hearing words/sentences) may occur with temporal lobe activity. Therefore, VE should be considered in a patient with episodic dizziness complaints and a family history of seizures.

### Work-up

There are no established criteria for diagnosing patients with VE. Diagnosis therein relies on a detailed description of episodes, detecting abnormal sleep-deprived electroencephalography (EEG) findings, and ruling out other causes of the origin of epileptic discharge. History-taking should attempt to characterize the vestibular and associated symptoms, including motor activity, with a focus on the episode’s duration, possible triggers, and loss of consciousness (LOC). Physical examination may reveal central findings on oculomotor testing such as abnormal saccades, spontaneous or dynamically-provoked nystagmus that does not fatigue nor suppress with fixation, or abnormal smooth pursuit. An MRI with gadolinium contrast may be ordered to rule out structural etiologies related to symptoms (e.g., infarct, tumor) but is rarely performed (6), likely due to reported low diagnostic yield in the work-up of pediatric vertigo and the challenges of ordering such imaging (e.g., cost, required sedation in younger children) (8, 13). Any patients with LOC should undergo a cardiac work-up, including an electrocardiogram (EKG), an echocardiogram (ECHO), 24 h Holter monitoring, orthostatic vital sign measure, or tilt table testing. Vestibular function testing may rule out peripheral vestibular dysfunction if suspected on clinical history (i.e., episodic positional vertigo lasting seconds to minutes) and/or bedside exam (e.g., positive Romberg). However, abnormal findings on these tests have been reported in children with VE, possibly secondary to the effects of anti-seizure medications (ASM) on the peripheral vestibular system (36). As such, a thorough medication history should screen for off-label use of ASMs in the patient without known epilepsy (e.g., migraine). Otologic examination and audiologic testing are expected to be unremarkable in VE but may be warranted if the patient reports any ear-related symptoms other than vertigo, such as otalgia, otorrhea, or hearing disturbance. Laboratory testing is typically ordered to rule out any metabolic disturbance that may elicit seizures, such as hyponatremia. Genetic testing may be warranted with appropriate counseling if a family history is positive for epilepsy.

### EEG findings

One would expect interictal EEG in patients with VE to reveal epileptic discharges within the temporal–parietal-occipital regions, given their participation in the processing of vestibular input (Figure 1) (6, 37, 38). However, Currie and colleagues reported that only 19% of patients with temporal lobe epilepsy presented with vertiginous symptoms alone (39). Indeed, in their retrospective review of 190 pediatric stereoelectroecenphalographies (SEEGs), Taussig and colleagues found that electrical stimulation of the precuneus, the insular region, the anterior temporal operculum, and the retro-insular region/parietal operculum elicited vertiginous symptoms (40). Vestibular symptoms arising from electrical activity within multiple cortical areas underscore current theories supporting the vestibular system’s widespread, multi-sensory cortical representation (28). Additionally, as epileptiform discharge may escape detection during routine scalp EEG (41), especially given the brief duration of vertiginous symptoms, 24 h EEG can be considered to establish the diagnosis before starting ASM.

### Epileptic nystagmus

Epileptic nystagmus (EN), defined as rapid, involuntary repetitive eye movements, is rare as a sole finding in the setting of VE (42). Case reports of EN in the pediatric population report the nystagmus as predominately binocular and horizontal, with the rapid phase of nystagmus contralateral to the epileptic focus noted on EEG. Ictal discharges have been reported in temporal–parietal-occipital regions, likely related to their role in coordinating smooth-pursuit or saccadic eye movements (43, 44).

### Vestibulogenic seizures

The term “vestibulogenic epilepsy” refers to seizures evoked by vestibular stimuli and is a separate entity from VE (45, 46). Vestibulogenic seizures are a rare, idiopathic subtype of sensory-evoked epilepsies, also known as reflex epilepsies (47). Proposed pathophysiology involves aberrant afferent peripheral vestibular stimuli or improper central processing of this input. This leads to generalized epileptic activity via the reticular activating system in the brainstem and thalamocortical pathways (45). Kogeorgos et al. (35) found that approximately 23% of all vestibulogenic seizures manifest as general tonic–clonic seizures. While simultaneous abnormalities on VNG caloric testing and ictal EEG would be expected to make the diagnosis of vestibulogenic epilepsy, clinicians would also need to exclude confounding variables (e.g., existing peripheral vestibular disorder, the effect of concurrent ASMs, or other medications).

### Differentiating VE from other non-epileptic vestibular disorders

Given its lack of established diagnostic criteria, VE must be delineated from more common vestibular disorders and vice versa. More than half of children presenting with dizziness also complain of headache (48, 49), so it is no surprise that Recurrent Vertigo of Childhood (RVC) (formerly, Benign Paroxysmal Vertigo of Childhood) and Vestibular Migraine of Childhood (VMC) often lead the diagnostic differential. VE a BPVC can be largely differentiated from VMC by the patient having a current or prior history of migraine and episodes of dizziness accompanied by migraine features (50). The presence of prodromal epileptic phenomena (e.g., visual or auditory aura or hallucinations, impaired consciousness) should alert the clinician toward VE, wherein vestibular symptoms are typically shorter (i.e., a few seconds) compared to RVC and VMC. Additionally, VE may mimic vestibular paroxysms, although the latter may yield horizontal and torsional nystagmus beating toward the affected ear and time-locked to the vestibular episode (51). Response to carbamazepine/oxcarbazepine treatment is also part of the vestibular paroxysmia diagnostic criteria. While an ideal specific test for VE would involve observing SEEG signals during episodes and mapping them to the vestibular cortical network via corticocortical-evoked potential technique (25), this would not be clinically feasible in routine work-up.

## Treatment

There is no consensus regarding first-choice ASMs in the treatment of VE in either the adult or pediatric population, as the evidence is limited to single case reports and small, heterogeneous cohort studies (1, 13, 30, 35, 52, 53). In one systematic review of 1,055 patients with VE [described as “epileptic vertigo or dizziness (EVD)”], the most commonly used ASMs were phenytoin, carbamazepine, and valproate, in descending order (6). Overall, patients with VE respond well to ASMs (14), with drug response rates reported as high as 90% (6). Of note, ASMs have also effectively treated other vestibular disorders of central origin, such as vestibular paroxysmia and vestibular migraine (54, 55).

The 2004 American Academy of Neurology guidelines for the treatment of new-onset focal or generalized epilepsies were updated, but limited recommendations in children to absence seizures (56) Thus, in approaching the treatment for suspected VE, the choice of ASM should be made first by classification of seizure type (focal vs. generalized) and then epilepsy type, which combines EEG data with the seizure semiology (symptoms and observed clinical manifestations). Once the type of epilepsy is determined, the choice of anti-seizure medication should consider the patient’s unique medical history, including active medications, co-morbidities, potential ASM side effects, required laboratory monitoring, and medication formulation.

Many children with epilepsy are unable to swallow pills or tablets, and thus, liquid medication should be utilized. In general, the newer generation ASMs (e.g., lamotrigine, levetiracetam) are better tolerated and have fewer side effects than the first-generation ASMs (e.g., phenobarbital, valproic acid). Clinicians should also be aware that medications such as valproate have been associated with peripheral vestibular function abnormalities on testing, such as electro/video-nystagmography (ENG/VNG) and vestibular-evoked myogenic potential responses (VEMP) (36). ASMs can also cause dizziness, so the side effect profile of the medical regimen should be carefully monitored.

## Conclusion

Vertiginous epilepsy (VE) is a specific type of epilepsy characterized by seizures with vestibular symptoms as the most prominent or even sole feature. Despite an increased prevalence in children compared to adults, diagnosis is often delayed and challenging due to the non-specific characterization of vestibular symptoms and the unique spread of electroactivity throughout the multi-sensory vestibular cortical network. Although there are no diagnostic criteria for VE, a thorough evaluation (detailed history, physical examination, MRI, EEG) to rule out structural causes of epilepsy and functional peripheral vestibulopathy is recommended for any pediatric patient presenting with vestibular symptoms accompanied by loss of consciousness. Reassuringly, children with VE generally respond well to ASMs and have a favorable prognosis.

### Future directions

- VE is rare, but diagnostic criteria should be developed to guide clinicians’ work-ups of children from other common causes of pediatric dizziness, including BPVC and vestibular migraine.
- Further research is needed to identify specific biomarkers or clinical characteristics that differentiate VE from other causes of vertigo in children.
- Studies are warranted to investigate the most effective treatment strategies and long-term outcomes for children with VE.

## Author contributions

AMW: Conceptualization, Writing – original draft, Writing – review & editing. AT-H: Conceptualization, Writing – original draft, Writing – review & editing. BWK: Supervision, Writing – review & editing.
